# Intratumoral and peritumoral ultrasound-based radiomics for preoperative prediction of HER2-low breast cancer: a multicenter retrospective study

**DOI:** 10.1186/s13244-025-01934-6

**Published:** 2025-03-07

**Authors:** Siwei Luo, Xiaobo Chen, Mengxia Yao, Yuanlin Ying, Zena Huang, Xiaoya Zhou, Zuwei Liao, Lijie Zhang, Na Hu, Chunwang Huang

**Affiliations:** 1 Department of Ultrasound, Guangdong Provincial People’s Hospital (Guangdong Academy of Medical Sciences), Southern Medical University, Guangzhou, China; 2 Department of Radiology, Guangdong Provincial People’s Hospital (Guangdong Academy of Medical Sciences), Southern Medical University, Guangzhou, China; 3 Guangdong Provincial Key Laboratory of Artificial Intelligence in Medical Image Analysis and Application, Guangzhou, China; 4 Department of Ultrasound, The First Affiliated Hospital of Guangzhou University of Traditional Chinese Medicine, Guangzhou University of Traditional Chinese Medicine, Guangzhou, China; 5 Department of Ultrasound, Sun Yat-Sen Memorial Hospital, Sun Yat-sen University, Guangzhou, China; 6 Department of Ultrasound, The People’s Hospital of Shangyou County, Ganzhou, China

**Keywords:** Invasive breast cancer, HER2-low, Ultrasonography, Machine learning, Peritumoral radiomics

## Abstract

**Objectives:**

Recent advances in human epidermal growth factor receptor 2 (HER2)-targeted therapies have opened up new therapeutic options for HER2-low cancers. This study aimed to establish an ultrasound-based radiomics model to identify three different HER2 states noninvasively.

**Methods:**

Between May 2018 and December 2023, a total of 1257 invasive breast cancer patients were enrolled from three hospitals. The HER2 status was divided into three classes: positive, low, and zero. Four peritumoral regions of interest (ROI) were auto-generated by dilating the manually segmented intratumoral ROI to thicknesses of 5 mm, 10 mm, 15 mm, and 20 mm. After image preprocessing, 4720 radiomics features were extracted from each image of every patient. The least absolute shrinkage and selection operator and LightBoost algorithm were utilized to construct single- and multi-region radiomics signatures (RS). A clinical–radiomics combined model was developed by integrating discriminative clinical-sonographic factors with the optimal RS. A data stitching strategy was used to build patient-level models. The Shapley additive explanations (SHAP) approach was used to explain the contribution of internal prediction.

**Results:**

The optimal RS was constructed by integrating 12 tumor features and 9 peritumoral-15mm features. Age, tumor size, and seven qualitative ultrasound features were retained to construct the clinical–radiomics combined model with the optimal RS. In the training, validation, and test cohorts, the patient-level combined model showed the best discrimination ability with the macro-AUCs of 0.988 (95% CI: 0.983–0.992), 0.915 (95% CI: 0.851–0.965), and 0.862 (95% CI: 0.820–0.899), respectively.

**Conclusion:**

This study built a robust and interpretable clinical–radiomics model to evaluate three classes of HER2 status based on ultrasound images.

**Critical relevance statement:**

Ultrasound-based radiomics method can noninvasively identify three different states of HER2, which may guide treatment decisions and the implementation of personalized HER2-targeted treatment for invasive breast cancer patients.

**Key Points:**

- Determination of HER2 status can affect treatment options for breast cancer.
- The ultrasound-based clinical–radiomics model can discriminate the three different HER2 statuses.
- Our developed model can assist in providing personalized recommendations for novel HER2-targeted therapies.

**Graphical Abstract:**

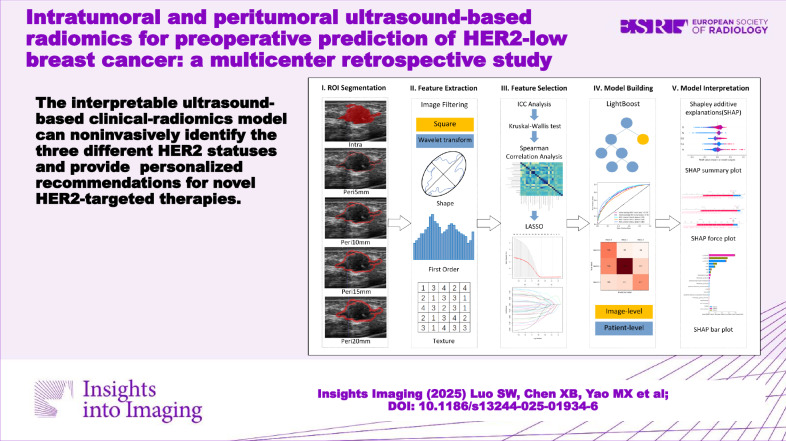

## Introduction

Breast cancer is one of the most common cancers diagnosed in women, accounting for 31% of female cancers [1]. The biological and clinical relevance of human epidermal growth factor receptor 2 (HER2) expression in breast cancer has had a major impact on the treatment of this disease [2]. The dichotomous definition of HER2 positive vs. negative is currently experiencing a wave of changes by including the identification of the “HER2-low” category for identifying patients eligible for treatment with trastuzumab deruxtecan. This has been approved by the US Food and Drug Administration and the European Medicines Agency, based on the promising results of the phase III DESTINY-Breast04 clinical trial [3–6]. The current assessment of HER2 based on immunohistochemistry (IHC) and fluorescence in situ hybridization (FISH) performed on breast cancer tissues obtained through biopsies or surgeries is expensive, invasive, time-consuming, and lagging [7, 8]. Moreover, the static evaluation at the one-time point may lead to an inevitable sampling bias, and hardly represent the heterogeneity and dynamic evolution of HER2 status during disease progression and neoadjuvant chemotherapy [3, 9, 10]. Therefore, it is necessary to construct a dynamic, global, and noninvasive marker to evaluate HER2 status.

Imaging biomarkers can assess and monitor HER2 status noninvasively and at zero cost using existing image data without additional tests by capturing information relating to an entire tumor, as well as its surrounding environment [10, 11]. Compared with other imaging modalities, ultrasound has several unique advantages, including low cost, real-time evaluation, wider availability and portability, no requirement for contrast administration, and a lack of ionizing radiation [12]. Radiomics characterizes tumor properties at the macro-scales through the high-throughput generation and the interrogation of massive quantitative features from digital images [13, 14]. Several previous studies have demonstrated the feasibility of using radiomics methods to predict HER2-low status in breast cancer based on ultrasound images. It may be noted that these studies mainly focused on distinguishing HER2-zero from HER2-low, or HER2-zero from HER2-low and -positive [15–17], had relatively small datasets [15–17], and lacked independent external validation cohorts [17]. The peritumoral region may embed biological information about tumor growth, invasion, and metastasis and may be a potential biomarker [18]. Several studies have indicated the importance of peritumoral features not only for distinguishing HER2-positive from HER2-negative [19–21], but also for distinguishing HER2-low from HER2-zero [22]. However, to date, no study has focused on the peritumoral radiomics features in ultrasound images to predict the three categories of HER2 expression.

In this study, we first explored the optimal peritumoral region for evaluating the three different states of HER2 and constructed a multi-region radiomics signature (RS) that integrates ultrasound tumor heterogeneity and optimal peritumoral microenvironment information. Furthermore, by incorporating the ultrasound RS and important clinical-sonographic predictors, an interpretable machine-learning model was developed. Finally, we investigated the model’s ability to differentiate the three different states of HER2 at both image level and patient level, which would help clinicians to better classify patients for precise therapeutic care.

## Materials and methods

### Study population

**Fig. 1:**
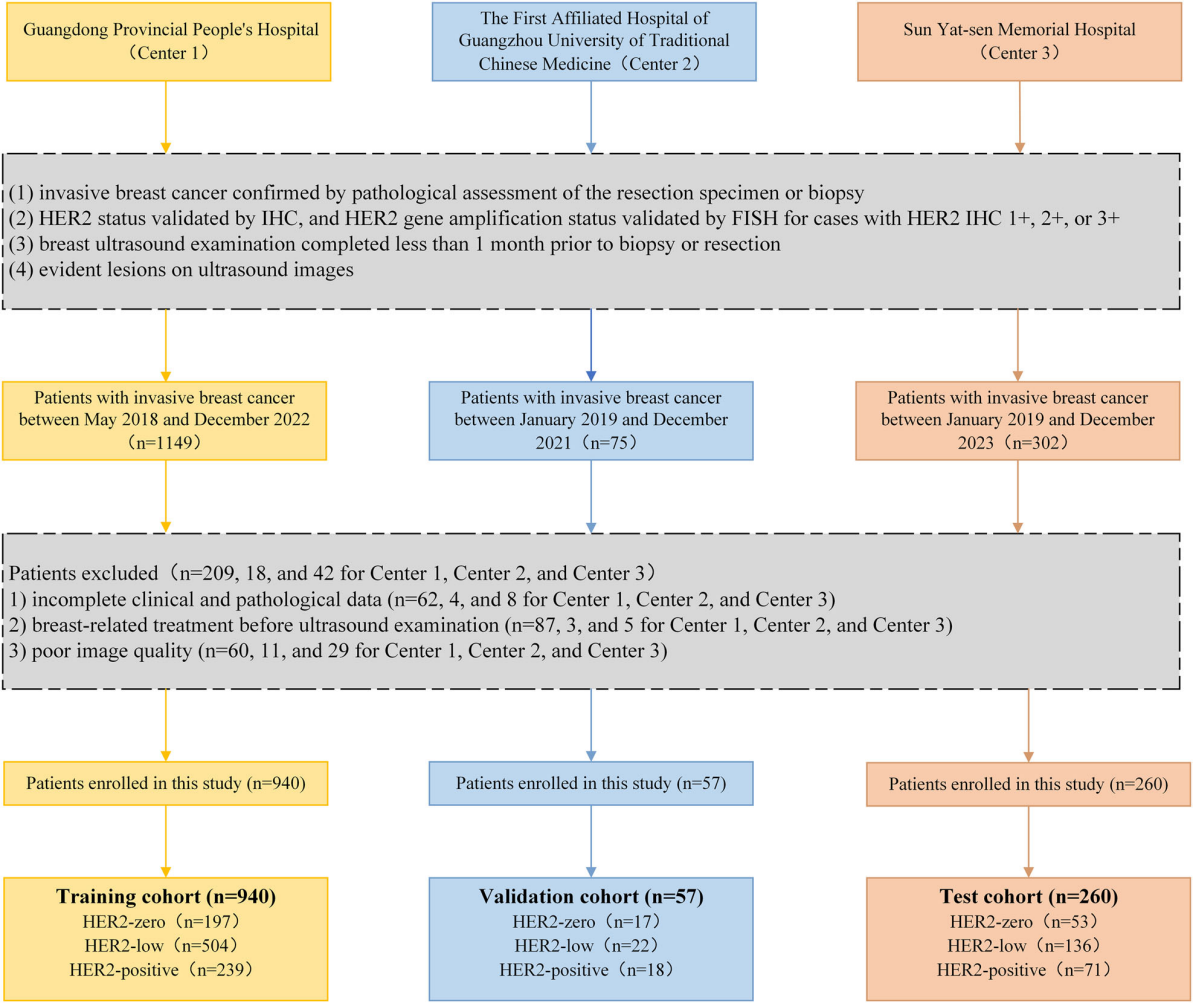
Flowchart of patient inclusion and exclusion criteria for eligible patients in the study. IHC, immunohistochemistry; FISH, fluorescence in situ hybridization

A total of 1149, 75, and 302 patients were pathologically diagnosed with invasive breast cancer from Guangdong Provincial People’s Hospital (Center 1, between May 2018 and December 2022), the First Affiliated Hospital of Guangzhou University of Traditional Chinese Medicine (Center 2, between January 2019 and December 2021) and Sun Yat-Sen Memorial Hospital (Center 3, between January 2019 and December 2023), respectively, were retrospectively enrolled (Fig. 1). This study was approved by the institutional review boards of the three centers, and the requirement for informed consent was waived. This study complied with the principles of the Declaration of Helsinki. The inclusion criteria were as follows: (1) invasive breast cancer confirmed by pathological assessment of the resection specimen or biopsy; (2) HER2 status validated by IHC, and HER2 gene amplification status validated by FISH for cases with HER2 IHC 1+, 2+, or 3+; (3) breast ultrasound examination completed less than 1 month prior to biopsy or resection; and (4) evident lesions on ultrasound images. The exclusion criteria were as follows: (1) incomplete clinical and pathological data; (2) breast-related treatment, such as chemotherapy, radiotherapy, or hormone therapy, before ultrasound examination; and (3) poor image quality (e.g., motion and speed propagation, refraction artifacts, or low-resolution images). Based on the exclusion criteria, 269 patients were excluded because of incomplete clinical and pathological data (*n* = 74), breast-related treatment before ultrasound examination (*n* = 95), and poor image quality (*n* = 100). In all, 3504 ultrasound images from Center 1 (940 women), 195 from Center 2 (57 women), and 652 from Center 3 (260 women) were allocated to the training, validation, and test cohorts, respectively.

### Clinicopathological data

Medical records and pathological reports of the resected specimens were reviewed to collect clinical information (age, tumor position, and presence of distant metastases) and pathological data (histological type, histologic grade, estrogen receptor (ER) status, progesterone receptor (PR) status, HER2 status, Ki-67 index, and lymph node metastases). The expression of the ER, PR, HER2, and Ki-67 was detected using IHC or FISH, according to the ASCO/CAP guidelines [4, 8, 23]. When at least 1% of the tumor cell nuclei stained positive for ER or PR, the samples were considered ER-positive or PR-positive, respectively. A value of 20% was taken as the cut-off point for the Ki-67 index assessment. For HER2 expression, patients were classified into three categories based on the scores obtained from IHC and FISH: (1) HER2-positive if a HER-2 staining intensity score of 3+ or 2+ plus positive FISH was obtained; (2) HER2-low if a score of 1+ or 2+ plus negative FISH was obtained; and (3) HER2-zero if a score of 0 was obtained [8, 24].

### Ultrasound examination

Breast ultrasound images were acquired using fifteen different devices (Supplementary Table S1). Qualitative ultrasound features, including tumor size, shape, orientation, margin, echo pattern, posterior features, calcifications, edema, skin changes, vascularity, and axillary lymph node metastasis (ALNM), were evaluated by two radiologists (Reader 1, Y.Y. and Reader 2, Z.H., both with 5 years of experience in breast ultrasound) according to the Breast Imaging Reporting and Data System, fifth edition. In cases of disagreement, Reader 3 (C.H. with 20 years of experience) made the final decision. The readers were blinded to the clinical and pathological information of the patients. The ultrasound assessment criteria for ALNM are provided in Supplementary S1.

### Image segmentation and feature extraction

**Fig. 2:**
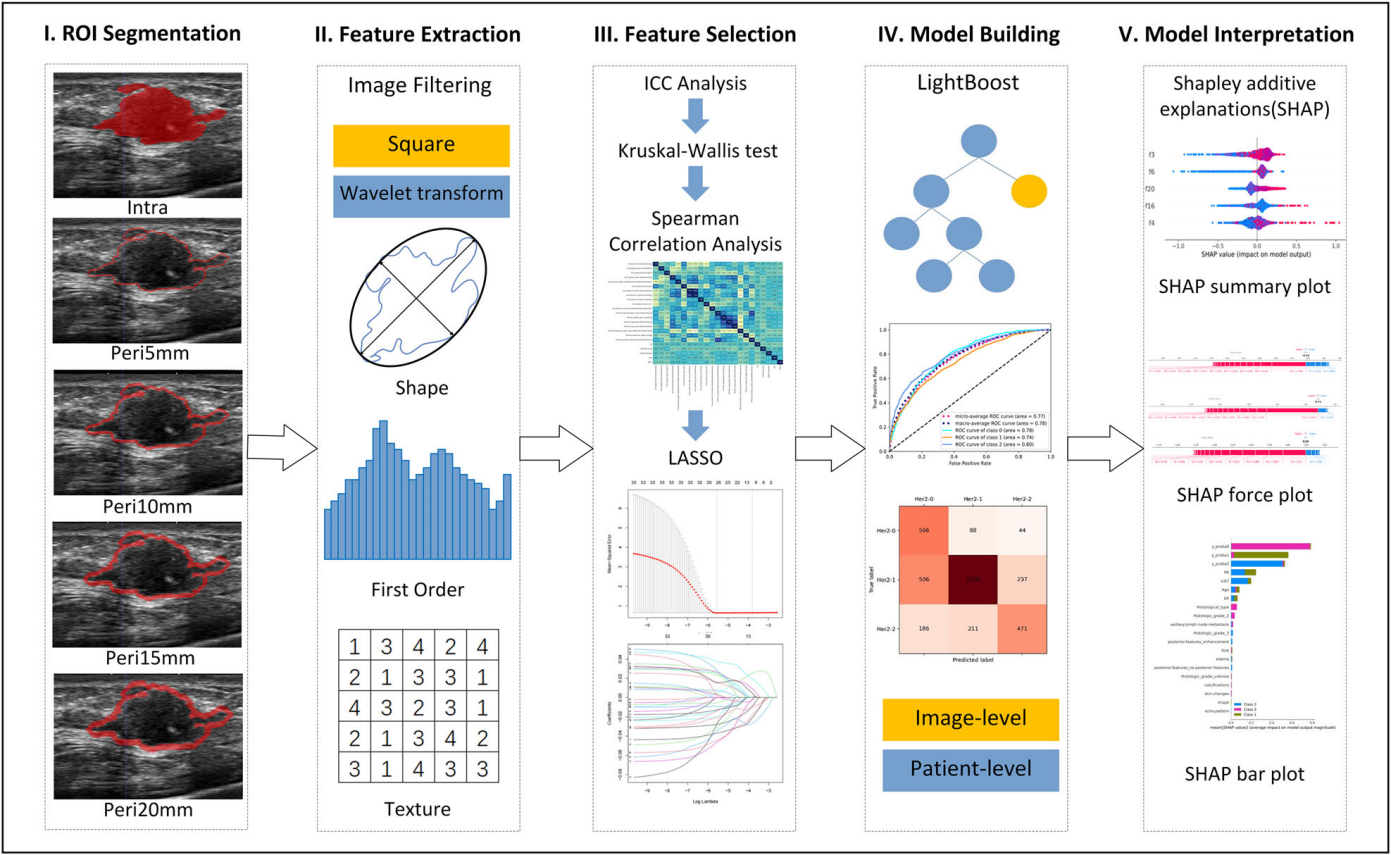
Overview of the radiomics analysis workflow. I Intratumoral and peritumoral ROIs segmentation on ultrasound images. II Radiomics features extraction from ultrasound images. III A series of coarse-to-fine feature selection strategies were carried out for feature selection. IV Construction and validation of models at image- and patient-level: LightBoost algorithm was used to build models; ROC curves and confusion matrix were implemented to assess the models’ performance. V The SHAP technique was used to visually explain the prediction process of models. ROI, region of interest; ICC, intraclass correlation coefficient; LASSO, the least absolute shrinkage and selection operator; ROC, receiver operating characteristic; SHAP, Shapley additive explanations

The intratumoral region of interest (ROI) was manually outlined along the contour of the tumor on ultrasound images by Reader 4 (S.L. with 10 years of experience in breast ultrasound) using the ITK-SNAP software (version 3.8.0; www.itksnap.org). Reader 4 and Reader 5 (M.Y. also with 10 years of experience in breast ultrasound) performed manual independent segmentation of the ROI from 30 randomly chosen patients to evaluate intra- and inter-observer reliability. Radiologists reviewed the raw images to confirm the tumor position and were blinded to the pathological outcomes and clinical messages. For multifocal lesions, only the largest mass was used for image segmentation and analysis. All images and tumor ROI were resampled to a voxel size of 1 × 1 × 1 mm^3^ using a linear interpolation method to standardize the voxel spacing. Next, the peritumoral ROIs of 5 mm, 10 mm, 15 mm, and 20 mm were auto-generated with the “SimpleITK” package using Python version 3.7. If the contours of the peritumoral regions extended beyond the breast parenchyma after expansion, the portion beyond the parenchyma was manually removed. Image preprocessing methods included the original image, square, and wavelet transform. For each ROI per image, 944 features from seven feature classes were extracted using the Pyradiomics package (version 2.1.0; https://pyradiomics.readthedocs.io/) in Python version 3. 7 (Supplementary S2). Because there were five ROIs (Tumor, Peri5mm, Peri10mm, Peri15mm, and Peri20mm), a total of 4720 radiomics features were extracted from each image for each patient simultaneously. The overall workflow of this study is summarized in Fig. 2.

### Feature selection

The random oversampling technique was adopted to alleviate model bias towards the majority class. Considering that the issue of different instruments being used in different centers is significant, we used the *Z*-score and ComBat method to reduce the batch effects. Several feature selection methods were used to further reduce overfitting and improve the robustness of the model. First, the intraclass correlation coefficient (ICC) was used to assess the robustness of the image features, and stable features with ICC > 0.75 were considered to have good consistency and selected for further analysis. Next, a Kruskal multigroup rank-sum test was performed to identify features that were significantly different across the three groups (*p* < 0.05). Subsequently, Spearman correlation analysis was used to assess the correlation between features and reduce redundancy among feature sets. Finally, we employed the least absolute shrinkage and selection operator (LASSO) regression method to select the optimized subset of radiomics features (Supplementary S3 and Fig. S1a, b). Features with non-zero coefficients were retained for further model building. To construct four multi-region radiomics models (Tumor + Peri5mm, Tumor + Peri10mm, Tumor + Peri15mm, and Tumor + Peri20mm), the above feature selection process was repeated using the radiomics features extracted from the intratumoral ROI with features generated by different peritumoral ROIs (Peri5mm, Peri10mm, Peri15mm, and Peri20mm, respectively) (Supplementary Fig. S1c, d).

### Construction of image-level multi-region ultrasound radiomics model

We employed the LightBoost algorithm to derive radiomics models. The hyperparameters were tuned by the GridSearch approach and five-fold cross verification and set as follows: ‘estimators’: 70, ‘depth’: 5, ‘learning_rate’: 0.15, ‘num_leaves’: 20. For a fair comparison, we fixed our test set, machine learning algorithm, and super parameters to the maximum extension possible. We constructed eight radiomics models, including five single-region radiomics models (Tumor, Peri5mm, Peri10mm, Peri15mm, and Peri20mm) and four multi-region radiomics models (Tumor + Peri5mm, Tumor + Peri10mm, Tumor + Peri15mm, and Tumor + Peri20mm). The optimal peritumoral region was determined to have the highest micro-average area under the receiver operating characteristic curve (AUC) and macro-average-AUC.

### Construction of image-level clinical–radiomics combined model

Based on the results of the Brant–Wald test, multinomial logistic regression analysis was conducted to evaluate the predictive value of clinical and qualitative sonographic factors after one-hot encoding on HER2 status. Important clinical-sonographic characteristics were selected with both *p* < 0.05 at uni- and multinomial logistic regression. The selected factors were then used to construct a clinical model using the LightBoost algorithm. Furthermore, a clinical–radiomics combined model was constructed by integrating discriminative clinical-sonographic factors and prediction probability scores calculated using the optimal RS.

### Construction of patient-level model

Owing to the retrospective nature of the study, the multi-view ultrasound image data were not completely preserved in all patients as each patient had a different number of ultrasound images. To make full use of all images from each patient and to mimic the workflow of radiologists who observe lesions from multi-view, we used a data stitching strategy to average the image-level prediction probability to form a final patient-level prediction probability score [25].

### Statistical analysis

All statistical analyses were performed using Python (v. 3.7.3) and R (v. 4.1.3) software. We conducted a comparison of the clinicopathological and qualitative ultrasound characteristics among the patients of three classes utilizing the Kruskal–Wallis test, analysis of variance (ANOVA), or χ^2^ test. Multinomial logistic regression analysis was used to analyze the association between the HER2 status and the clinical-sonographic features. The AUC and confusion matrix were used for the performance estimation. The accuracy, micro- and macro-AUC of the models were calculated at both the image and patient levels for the three-category classification task. Shapley additive explanations (SHAP) were used to investigate the relationship between HER2 status and modeling features. A two-tailed *p* < 0.05 was considered statistically significant.

## Results

### Patient characteristics

A total of 1257 patients from Center 1 (940 cases, 52.24 ± 11.50 years), Center 2 (57 cases, 51.30 ± 9.69 years), and Center 3 (260 cases, 53.40 ± 11.71 years) were included in this study. The detailed clinicopathological and qualitative ultrasound characteristics are described in Tables 1 and 2, respectively. HER2-low was the most common subtype, comprising 52.7% of the cases, HER2-zero was the least common accounting for 21.2% of the cases, while HER2-positive tumors accounted for 26.1% of the cases. PR- and ER-positive were more common in the HER2-zero and HER2-low groups, whereas the HER2-positive group had a larger tumor size, higher Ki-67 index, and a higher proportion of lymph node metastases and calcifications in the training and test cohorts. Histological type, histologic grade, orientation, and shape also showed significant differences among the three classes in the training cohort, whereas age and vascularity showed significant differences in the test cohort (*p* < 0.05).

**Table 1:** Clinical and pathological characteristics of patients grouped by HER2 status

Characteristics	Center 1 (*N* = 940)				Center 2 (*N* = 57)				Center 3 (*N* = 260)			
	HER2-zero (*N* = 197)	HER2-low (*N* = 504)	HER2-positive (*N* = 239)	*p*-value	HER2-zero (*N* = 17)	HER2-low (*N* = 22)	HER2-positive (*N* = 18)	*p*-value	HER2-zero (*N* = 53)	HER2-low (*N* = 136)	HER2-positive (*N* = 71)	*p*-value
Age^#^	51.64 ± 10.26	52.83 ± 12.00	51.43 ± 11.62	0.46^a^	48.29 ± 12.07	52.55 ± 7.95	52.61 ± 9.31	0.324^b^	50.75 ± 12.69	55.04 ± 12.03	52.20 ± 9.99	0.040^b,*^
Tumor position				0.866				0.657				0.805
Left	100 (50.8%)	253 (50.2%)	125 (52.3%)		9 (52.9%)	12 (54.5%)	12 (66.7%)		25 (47.2%)	71 (52.2%)	35 (49.3%)	
Right	97 (49.2%)	251 (49.8%)	114 (47.7%)		8 (47.1%)	10 (45.5%)	6 (33.3%)		28 (52.8%)	65 (47.8%)	36 (50.7%)	
Histological type				0.001*				0.276				0.088
NST	144 (73.1%)	412 (81.7%)	207 (86.6%)		15 (88.2%)	19 (86.4%)	18 (100%)		34 (64.2%)	99 (72.8%)	58 (81.7%)	
Other	53 (26.9%)	92 (18.3%)	32 (13.4%)		2 (11.8%)	3 (13.6%)	0 (0%)		19 (35.8%)	37 (27.2%)	13 (18.3%)	
Histologic grade				< 0.001*				0.06				0.092
I	17 (8.6%)	40 (7.9%)	4 (1.7%)		1 (5.9%)	5 (22.7%)	0 (0%)		0 (0%)	1 (0.7%)	0 (0%)	
II	104 (52.8%)	333 (66.1%)	135 (56.5%)		12 (70.6%)	12 (54.5%)	9 (50%)		23 (43.4%)	56 (41.2%)	21 (29.6%)	
III	63 (32%)	120 (23.8%)	91 (38.1%)		3 (17.6%)	5 (22.7%)	9 (50%)		20 (37.7%)	31 (22.8%)	26 (36.6%)	
Unknown	13 (6.6%)	11 (2.2%)	9 (3.8%)		1 (5.9%)	0 (0%)	0 (0%)		10 (18.9%)	48 (35.3%)	24 (33.8%)	
ER status				< 0.001*				0.096				< 0.001*
Negative	36 (18.3%)	52 (10.3%)	77 (32.2%)		5 (29.4%)	4 (18.2%)	9 (50%)		13 (24.5%)	12 (8.8%)	28 (39.4%)	
Positive	161 (81.7%)	452 (89.7%)	162 (67.8%)		12 (70.6%)	18 (81.8%)	9 (50%)		40 (75.5%)	124 (91.2%)	43 (60.6%)	
PR status				< 0.001*				0.013*				< 0.001*
Negative	54 (27.4%)	87 (17.3%)	113 (47.3%)		6 (35.3%)	8 (36.4%)	14 (77.8%)		13 (24.5%)	12 (8.8%)	28 (39.4%)	
Positive	143 (72.6%)	417 (82.7%)	126 (52.7%)		11 (64.7%)	14 (63.6%)	4 (22.2%)		40 (75.5%)	124 (91.2%)	43 (60.6%)	
ki67				< 0.001*				0.085				< 0.001*
< 20%	65 (33%)	170 (33.7%)	26 (10.9%)		4 (23.5%)	5 (22.7%)	0 (0%)		17 (32.1%)	30 (22.1%)	2 (2.8%)	
≥ 20%	132 (67%)	334 (66.3%)	213 (89.1%)		13 (76.5%)	17 (77.3%)	18 (100%)		36 (67.9%)	106 (77.9%)	69 (97.2%)	
lymph node involvement or metastases				0.001*				0.31				< 0.001*
Absent	121 (61.4%)	278 (55.2%)	106 (44.4%)		11 (64.7%)	11 (50%)	7 (38.9%)		43 (81.1%)	64 (47.1%)	38 (53.5%)	
Present	76 (38.6%)	226 (44.8%)	133 (55.6%)		6 (35.3%)	11 (50%)	11 (61.1%)		10 (18.9%)	72 (52.9%)	33 (46.5%)	
Distant metastases				0.429				0.521				0.964
Nonmetastasis	193 (98%)	485 (96.2%)	229 (95.8%)		16 (94.1%)	22 (100%)	17 (94.4%)		49 (92.5%)	125 (91.9%)	66 (93%)	
Metastasis	4 (2%)	19 (3.8%)	10 (4.2%)		1 (5.9%)	0 (0%)	1 (5.6%)		4 (7.5%)	11 (8.1%)	5 (7%)	

*NST* invasive carcinoma of no special type, *ER* estrogen receptor, *PR* progesterone receptor, *Ki-67* cellular proliferation index

^#^ Data are described as mean ± standard deviation. Other data are shown as numbers of patients and proportions with percentages in parentheses

* *p* < 0.05

^a^ *p*-values were calculated using the Kruskal–Wallis test

^b^ *p*-values were calculated using ANOVA. Other *p*-values were calculated using χ^2^ test

**Table 2:** Qualitative ultrasound characteristics of patients grouped by HER2 status

Characteristics	Center 1 (*N* = 940)				Center 2 (*N* = 57)				Center 3 (*N* = 260)			
	HER2-zero (*N* = 197)	HER2-low (*N* = 504)	HER2-positive (*N* = 239)	*p*-value	HER2-zero (*N* = 17)	HER2-low (*N* = 22)	HER2-positive (*N* = 18)	*p*-value	HER2-zero (*N* = 53)	HER2-low (*N* = 136)	HER2-positive (*N* = 71)	*p*-value
Size^#^	2.30 (1.70–3.00)	2.30 (1.70–3.20)	2.80 (2.10–3.70)	< 0.001^a,*^	2.96 ± 0.92	3.52 ± 1.56	3.64 ± 1.29	0.276^b^	1.00 (1.00–1.00)	2.90 (2.15–3.80)	3.20 (2.55–4.45)	< 0.001^a,*^
Shape				0.017*				0.445				0.471
Irregular	195 (99%)	490 (97.2%)	239 (100%)		17 (100%)	21 (95.5%)	18 (100%)		52 (98.1%)	132 (97.1%)	67 (94.4%)	
Regular	2 (1%)	14 (2.8%)	0 (0%)		0 (0%)	1 (4.5%)	0 (0%)		1 (1.9%)	4 (2.9%)	4 (5.6%)	
Orientation				0.019*				0.352				0.076
Not parallel	38 (19.3%)	109 (21.6%)	31 (13%)		2 (11.8%)	2 (9.1%)	0 (0%)		9 (17%)	22 (16.2%)	4 (5.6%)	
Parallel	159 (80.7%)	395 (78.4%)	208 (87%)		15 (88.2%)	20 (90.9%)	18 (100%)		44 (83%)	114 (83.8%)	67 (94.4%)	
Margin				0.194				0.302				0.946
Not circumscribed	187 (94.9%)	489 (97%)	234 (97.9%)		16 (94.1%)	22 (100%)	18 (100%)		51 (96.2%)	132 (97.1%)	69 (97.2%)	
Circumscribed	10 (5.1%)	15 (3%)	5 (2.1%)		1 (5.9%)	0 (0%)	0 (0%)		2 (3.8%)	4 (2.9%)	2 (2.8%)	
Echo pattern				0.11				0.074				0.916
Uniform	38 (19.3%)	111 (22%)	37 (15.5%)		1 (5.9%)	7 (31.8%)	2 (11.1%)		7 (13.2%)	21 (15.4%)	10 (14.1%)	
Heterogenous	159 (80.7%)	393 (78%)	202 (84.5%)		16 (94.1%)	15 (68.2%)	16 (88.9%)		46 (86.8%)	115 (84.6%)	61 (85.9%)	
Posterior features				0.388				0.354				0.252
Negative	48 (24.4%)	132 (26.2%)	55 (23%)		5 (29.4%)	4 (18.2%)	1 (5.6%)		23 (43.4%)	69 (50.7%)	31 (43.7%)	
Enhancement	37 (18.8%)	63 (12.5%)	40 (16.7%)		1 (5.9%)	0 (0%)	2 (11.1%)		16 (30.2%)	22 (16.2%)	12 (16.9%)	
Shadowing	86 (43.7%)	228 (45.2%)	104 (43.5%)		5 (29.4%)	11 (50%)	7 (38.9%)		12 (22.6%)	33 (24.3%)	19 (26.8%)	
Combined pattern	26 (13.2%)	81 (16.1%)	40 (16.7%)		6 (35.3%)	7 (31.8%)	8 (44.4%)		2 (3.8%)	12 (8.8%)	9 (12.7%)	
Calcifications				< 0.001*				0.201				0.035*
Negative	68 (34.5%)	138 (27.4%)	35 (14.6%)		6 (35.3%)	12 (54.5%)	5 (27.8%)		34 (64.2%)	78 (57.4%)	30 (42.3%)	
Positive	129 (65.5%)	366 (72.6%)	204 (85.4%)		11 (64.7%)	10 (45.5%)	13 (72.2%)		19 (35.8%)	58 (42.6%)	41 (57.7%)	
Edema				0.512				0.769				0.122
Negative	79 (40.1%)	202 (40.1%)	106 (44.4%)		4 (23.5%)	4 (18.2%)	5 (27.8%)		27 (50.9%)	48 (35.3%)	31 (43.7%)	
Positive	118 (59.9%)	302 (59.9%)	133 (55.6%)		13 (76.5%)	18 (81.8%)	13 (72.2%)		26 (49.1%)	88 (64.7%)	40 (56.3%)	
Skin changes				0.073				0.605				0.099
Negative	182 (92.4%)	434 (86.1%)	210 (87.9%)		16 (94.1%)	19 (86.4%)	15 (83.3%)		52 (98.1%)	120 (88.2%)	65 (91.5%)	
Positive	15 (7.6%)	70 (13.9%)	29 (12.1%)		1 (5.9%)	3 (13.6%)	3 (16.7%)		1 (1.9%)	16 (11.8%)	6 (8.5%)	
Vascularity				0.075				0.12				0.021*
Absent	18 (9.1%)	51 (10.1%)	13 (5.4%)		0 (0%)	3 (13.6%)	0 (0%)		9 (17%)	6 (4.4%)	3 (4.2%)	
Internal vascularity	155 (78.7%)	402 (79.8%)	209 (87.4%)		16 (94.1%)	19 (86.4%)	18 (100%)		36 (67.9%)	114 (83.8%)	57 (80.3%)	
Vessels in rim	24 (12.2%)	51 (10.1%)	17 (7.1%)		1 (5.9%)	0 (0%)	0 (0%)		8 (15.1%)	16 (11.8%)	11 (15.5%)	
ALNM				< 0.001*				0.13				< 0.001*
Negative	121 (61.4%)	299 (59.3%)	100 (41.8%)		8 (47.1%)	9 (40.9%)	3 (16.7%)		36 (67.9%)	52 (38.2%)	26 (36.6%)	
Positive	76 (38.6%)	205 (40.7%)	139 (58.2%)		9 (52.9%)	13 (59.1%)	15 (83.3%)		17 (32.1%)	84 (61.8%)	45 (63.4%)	

*ALNM* axillary lymph node metastasis

^#^ Data are described as a median and interquartile range in the Centers 1 and 3 cohorts, and mean ± standard deviation in the Center 2 cohort. Other data are shown as proportions with percentages in parentheses

* *p* < 0.05

^a^ *p*-values were calculated using the Kruskal–Wallis test

^b^ *p*-values were calculated using ANOVA. Other *p*-values were calculated using χ^2^ test

### Construction and interpretability analysis of the optimal multi-region radiomics model

**Fig. 3:**
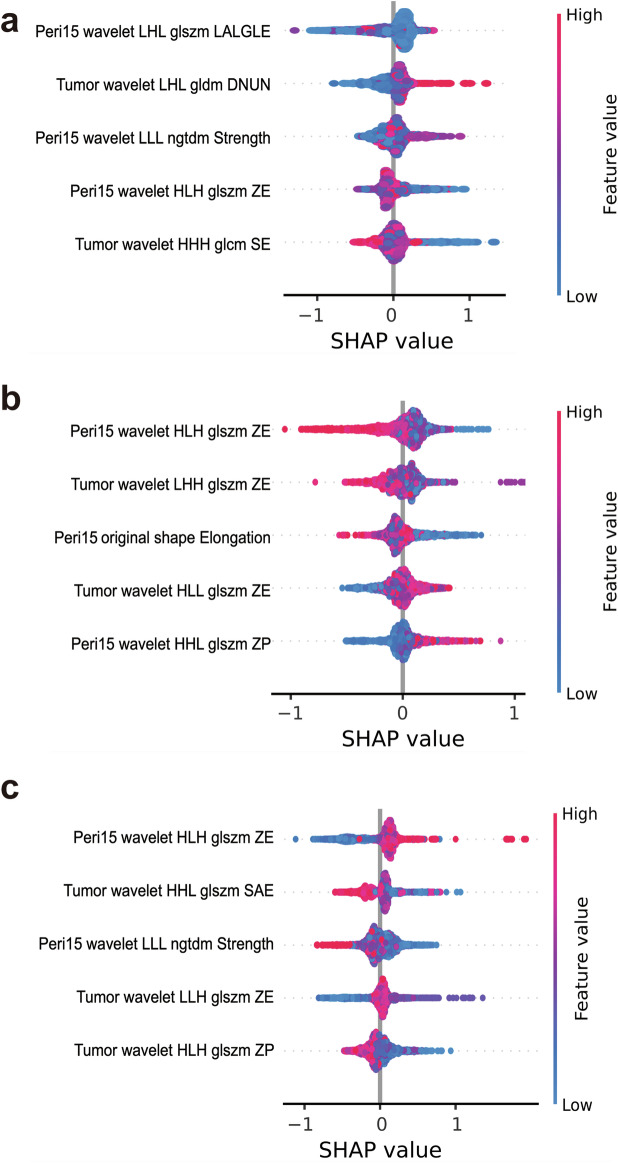
SHAP summary plot of Tumor + Peri15mm radiomics model. The SHAP summary plot shows the impact of the top five features on the model predictions for different HER2 classes. Individual dots symbolize patients, and different colors represent different levels of influence on the model output. **a** Prediction for HER2-zero class. **b** Prediction for HER2-low class. **c** Prediction for HER2-positive class. SHAP, Shapley additive explanations; LALGLE, large area low gray level emphasis; DNUN, dependence non-uniformity normalized; ZE, zone entropy; SE, sum entropy; ZP, zone percentage; SAE, small area emphasis

**Fig. 4:**
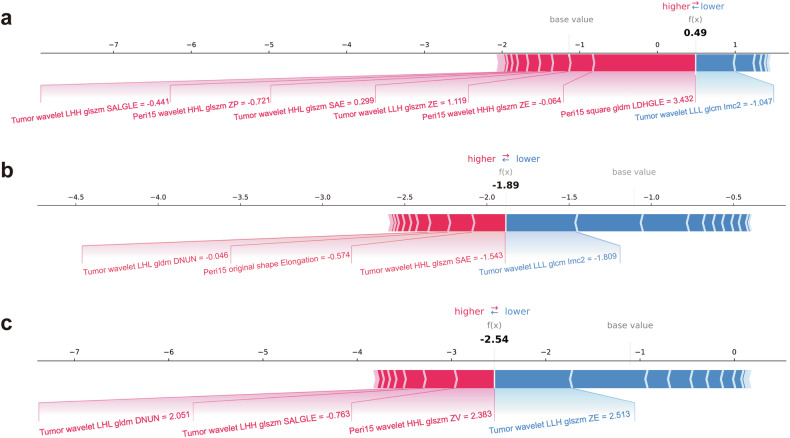
Application analysis of Tumor + Peri15mm radiomics model for three representative patients with different HER2 status. **a** HER2-zero patient. **b** HER2-low patient. **c** HER2-positive patient. SALGLE, small area low gray level emphasis; ZP, zone percentage; SAE, small area emphasis; ZE, zone entropy; LDHGLE, large dependence high gray level emphasis; DNUN, dependence non-uniformity normalized; ZV, zone variance

The performance of the Tumor and four multi-region radiomics models is displayed in Supplementary Fig. S2. The Tumor + Peri15mm model demonstrated the best performance with the macro-AUC and accuracy of 0.891 (95% CI: 0.882–0.899) and 70.2% (95% CI: 68.6–71.7%), 0.741 (95% CI: 0.686–0.793) and 57.4% (95% CI: 50.8–64.1%), and 0.746 (95% CI: 0.710–0.777) and 61.7% (95% CI: 58.0–65.2%) in the training, validation, and test cohorts, respectively. The detailed features of the Tumor + Peri15mm model are listed in Table 3. The SHAP summary plot shows the contributions of the top five features in each class (Fig. 3). Peri15mm_wavelet.LLL_ngtdm_Strength showed opposite impacts on HER2-zero and HER2-positive status, as well as Peri15mm_wavelet_HLH_glszm_ Zone Entropy (ZE) on HER2-zero, HER2-low, and HER2-positive status. The SHAP force plot can explain the evaluation of individual patients, and three representative patients with different HER2 statuses are shown in Fig. 4.

**Table 3:** Detailed radiomics features selected for establishing Tumor + Peri15mm model

Region	Filter	Type	Name
Tumor	wavelet.LLH	firstorder	Kurtosis
Tumor	wavelet.LLH	glszm	ZE
Tumor	wavelet.LHL	gldm	Dependence non-uniformity normalized
Tumor	wavelet.LHL	ngtdm	Strength
Tumor	wavelet.LHH	glszm	Small area low gray level emphasis
Tumor	wavelet.LHH	glszm	ZE
Tumor	wavelet.HLL	glszm	ZE
Tumor	wavelet.HLH	glszm	Zone percentage
Tumor	wavelet.HHL	glszm	Small area emphasis
Tumor	wavelet.HHH	glcm	Sum entropy
Tumor	wavelet.LLL	glcm	Cluster shade
Tumor	wavelet.LLL	glcm	Imc2
Peri-15mm	original	shape	Elongation
Peri-15mm	Square	gldm	Large dependence high gray level emphasis
Peri-15mm	wavelet.LHL	glszm	Large area low gray level emphasis
Peri-15mm	wavelet.HLH	glszm	ZE
Peri-15mm	wavelet.HHL	firstorder	Kurtosis
Peri-15mm	wavelet.HHL	glszm	Zone percentage
Peri-15mm	wavelet.HHL	glszm	Zone variance
Peri-15mm	wavelet.HHH	glszm	ZE
Peri-15mm	wavelet.LLL	ngtdm	Strength

### Construction and interpretability analysis of the clinical–radiomics models

Age, tumor size, and seven qualitative ultrasound features were selected to construct the clinical model (Supplementary Table S2). All features, with their degree of importance contributing to the prediction results, are shown in descending order in Supplementary Fig. S3a. The impact of the top five variables for predicting each classification of HER2 status is illustrated in the SHAP summary plot (Supplementary Fig. S3b–d). Several features had opposite influences on different predicted labels. Without calcifications in the tumor contributed to a HER2-zero outcome, and a smaller tumor size contributed to HER2-zero and HER2-low outcomes, whereas a larger tumor size and calcifications contributed to a high probability of HER2-positive. The prediction probability scores of the Tumor + Peri15mm radiomics model were the most important characteristics for each class when integrated with the feature set of the clinical model to construct a combined model (Supplementary Figs. S4 and S5).

### Image-level and patient-level model performance analysis

**Fig. 5:**
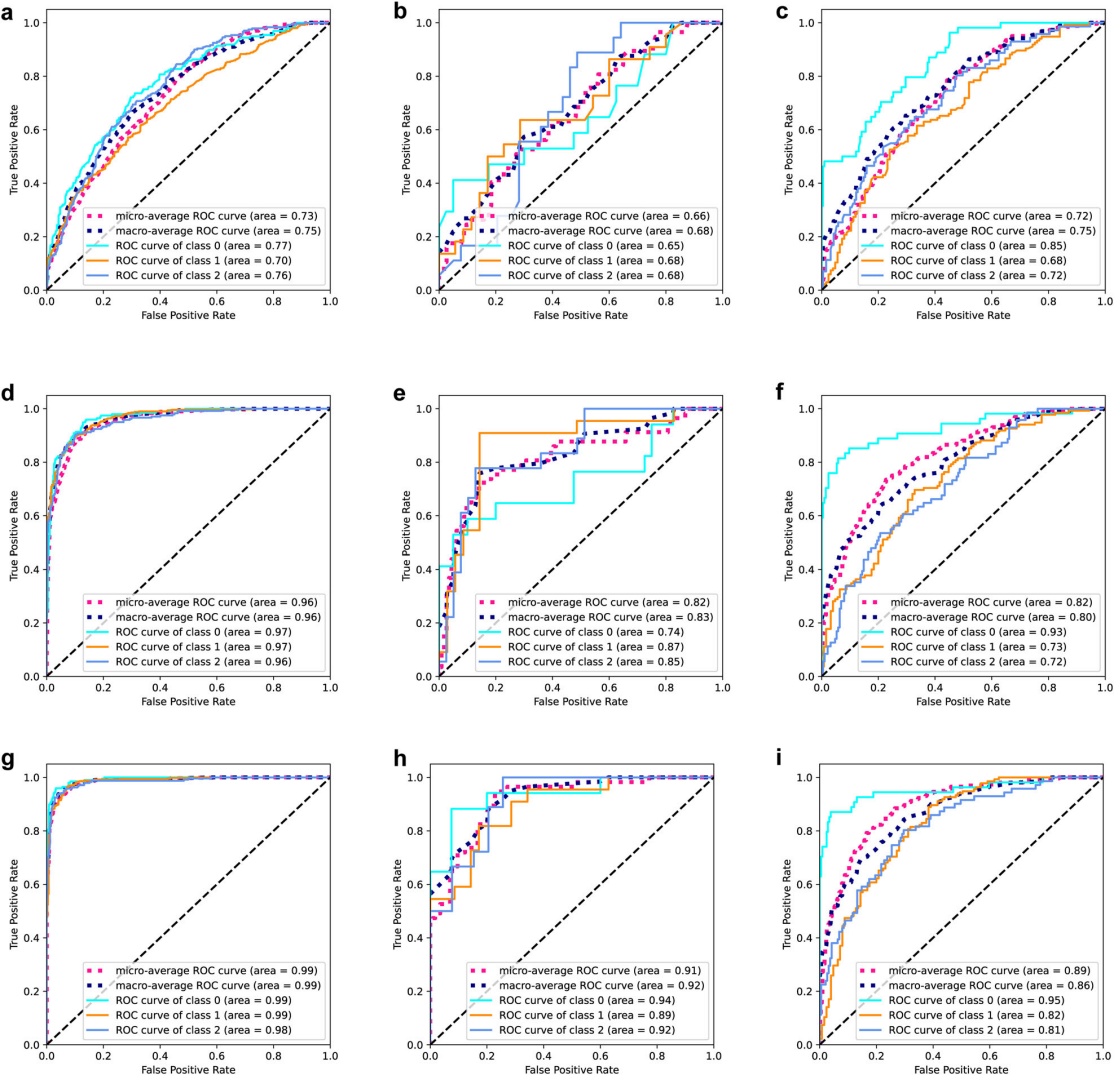
Performance of patient-level models in the training, validation, and test cohorts, respectively. **a**–**c** The ROC curves of the clinical model. **d**–**f** The ROC curves of the radiomics model. **g**–**i** The ROC curves of the combined model. 0 represents HER2-zero status, 1 represents HER2-low status, and 2 represents HER2-positive status. ROC, receiver operating characteristic

**Fig. 6:**
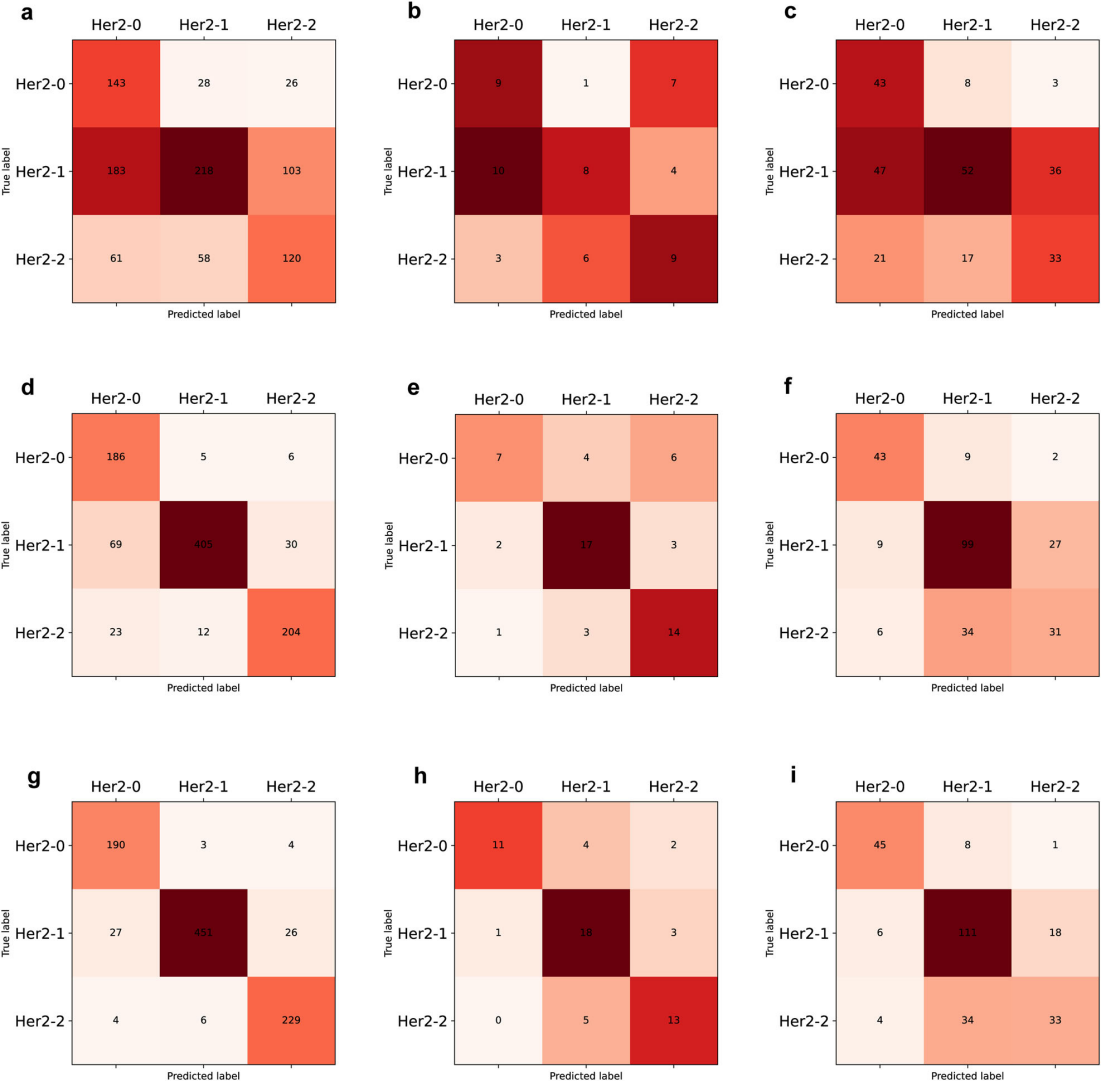
Confusion matrix of patient-level models in the training, validation, and test cohorts, respectively. **a**–**c** clinical model, (**d**–**f**) radiomics model, and (**g**–**i**) combined model. 0 represents HER2-zero status, 1 represents HER2-low status, and 2 represents HER2-positive status

The combined models showed the best discrimination ability to identify HER2 status at both the image and patient levels compared to the radiomics and clinical models alone (Table 4, Fig. 5, and Supplementary Fig. S6). Especially at the patient level, the combined model achieved the highest macro-AUC and accuracy of 0.988 (95% CI: 0.983–0.992) and 92.6% (95% CI: 90.9–94.0%), 0.915 (95% CI: 0.851–0.965) and 73.7% (95% CI: 61.4–84.2%), and 0.862 (95% CI: 0.820–0.899) and 72.7% (95% CI: 67.3–78.1%) in the training, validation, and test cohorts, respectively. The macro-AUC and accuracy of the radiomics model and combined model at the patient level showed better performance than the image-level models. This trend was not found in the clinical model, even the accuracy and macro-AUC of the patient level reduced slightly compared to the image level in the training and validation cohorts. Detailed model classification results were visualized using a confusion matrix (Fig. 6 and Supplementary Fig. S7).

**Table 4:** Discriminative performances of clinical, radiomics, and combined models in the training, validation, and test cohorts

Cohorts	Models	Image-level		Patient-level	
		macro-AUC (95% CI)	Accuracy (95% CI)	macro-AUC (95% CI)	Accuracy (95% CI)
Training	Clinical	0.768 (0.756, 0.779)	0.531 (0.514, 0.547)	0.745 (0.720, 0.767)	0.512 (0.483, 0.543)
	Radiomics	0.891 (0.882, 0.899)	0.702 (0.686, 0.717)	0.963 (0.954, 0.972)	0.846 (0.822, 0.867)
	Combined	0.946 (0.941, 0.952)	0.809 (0.795, 0.821)	0.988 (0.983, 0.992)	0.926 (0.909, 0.940)
Validation	Clinical	0.732 (0.680, 0.781)	0.533 (0.467, 0.600)	0.675 (0.562, 0.777)	0.456 (0.333, 0.579)
	Radiomics	0.741 (0.686, 0.793)	0.574 (0.508, 0.641)	0.828 (0.721, 0.906)	0.667 (0.526, 0.789)
	Combined	0.856 (0.811, 0.893)	0.667 (0.600, 0.728)	0.915 (0.851, 0.965)	0.737 (0.614, 0.842)
Test	Clinical	0.744 (0.715, 0.773)	0.491 (0.454, 0.526)	0.749 (0.705, 0.793)	0.492 (0.431, 0.554)
	Radiomics	0.746 (0.710, 0.777)	0.617 (0.580, 0.652)	0.796 (0.749, 0.836)	0.665 (0.608, 0.719)
	Combined	0.818 (0.788, 0.847)	0.686 (0.649, 0.721)	0.862 (0.820, 0.899)	0.727 (0.673, 0.781)

## Discussion

The emergence of novel HER2-targeted antibody–drug conjugates and other classes of drugs has brought revolutionary changes to the HER2-low breast cancer field [26]. In this multicenter study, we constructed an interpretable LightBoost clinical–radiomics combined model to distinguish the three different states of HER2. To our knowledge, this is the first attempt to use ultrasound-based intratumoral and peritumoral RSs. The individualized contribution of each feature was visualized for each patient using the Shapley algorithm, which helped explain the predictive power of the features in this model. Our developed models can offer a noninvasive approach to assessing HER2 levels in diverse individuals with breast malignancies and serve as a valuable tool to aid clinicians in making more precise and personalized decisions.

Like previous studies, the HER2-positive group had larger tumor volumes, more positive axillary lymph nodes, and a higher proliferation index. Moreover, a larger tumor volume showed a positive impact on the predicted probability of HER2-positive status but a negative impact on HER2-low and HER2-zero statuses. Overexpression of HER2 upregulates intrinsic kinase activity and activates several signaling cascades, which confers this subgroup of cancers with more aggressive behavior and worse prognosis [27, 28]. Among the three groups, the HER2-low group had the highest proportion of ER- and PR- positive statuses, which is consistent with previous reports [15, 29, 30]. Although HER2-low cancers can populate both luminal and non-luminal subtypes, they are typically hormone receptor-positive and preferentially pertain to the luminal molecular subgroup, according to gene expression analysis [3, 31, 32]. Furthermore, crosstalk between the HER2 receptor network and ER pathways is one of the proposed mechanisms for HER2 protein expression without underlying HER2 gene amplification [33].

Our results demonstrated that calcifications play a major positive role in predicting HER2-positive status and a negative role in predicting HER2-zero status, which is consistent with previous studies showing that calcification is more common in HER2-positive tumors and increases with HER2 IHC score [34–36]. In addition, edema and antiparallel orientation have a positive contribution to the HER2-low outcomes [15], but a negative contribution to the HER2-positive outcomes. These results may be attributed to the presence of triple-negative breast cancer within HER2-low cases, some of which are more frequently characterized by a round shape and perilesional edema [36, 37]. The twenty-one optimal radiomics features retained for modeling in our study were predominantly involved in tumor heterogeneity (Table 3). ZE was used to measure the uncertainty in the distribution of zone sizes and gray levels, and the SHAP summary plot showed that it was the most important feature variable for differentiating the three HER2 statuses (Fig. 3). A higher ZE value indicated a more heterogeneous voxel intensity in the ultrasound images and showed a positive impact on the predicted probability of a HER2-positive status, which is in agreement with previous studies [35, 38].

Currently, the morphological and radiomics analysis of HER2-low tumors mainly focuses on breast magnetic resonance imaging (MRI). In their studies, Bian et al and Guo et al first distinguished HER2-positive from HER2-negative (HER2-low and HER2-zero) breast cancers, and their models achieved AUCs of 0.760 to 0.763 in the validation cohort, further distinguishing HER2-low from HER2-zero breast cancers, AUCs of 0.711 to 0.750 were achieved in the validation cohort. The radiomics models of Zheng et al achieved AUCs of 0.725 for differentiating HER2-positive from others, 0.782 for differentiating HER2-low from others, and 0.813 for differentiating HER2-zero from others in the external validation datasets [39]. Our study contained a relatively larger dataset than other related studies, and the models we developed exhibited better differentiation capability for HER2 status trichotomy using ultrasound images, especially at the patient level.

Our study indicates that peritumoral features have potential predictive value and may contain additional information on HER2 expression, complementing the information provided by intratumoral features, which agrees with those previous peritumoral studies [19, 22, 40, 41]. The tumor microenvironment contains important biological information that may result in subtle changes in the images, such as angiogenesis, lymphangiogenic activity, peritumoral lymphatic and blood vessel invasion, peritumoral lymphocytic infiltration, peritumoral edema, and the release of peritumoral cytokines [19, 40, 42]. However, the optimal peritumoral area size for predicting the HER2 status from ultrasound images remains unclear. In the study conducted by Zhang et al, 8-mm was determined to be the optimal peritumoral size in the classification of HER2-enriched vs others on dynamic contrast-enhanced MRI [40]. In our study, the RS that relied on the intratumoral and peritumoral of the 15 mm ROIs showed good overall classification performance. This is probably because the annular peritumoral areas of 5 mm and 10 mm were too small to contain sufficient information, while the area around the lesion expanded to 20 mm and more potential normal breast tissue was included, which may have reduced the diagnostic efficacy [43].

This study has several limitations. First, the retrospective nature of the study, the variety of ultrasound machines, and parameter settings from different institutions may have influenced the performance of the radiomics models. In the future, a well-designed prospective cohort study is necessary to determine the effects of these possible confounding variables on the model performance. Second, the process of manual segmentation is labor-intensive, time-consuming, and relies on the knowledge and experience of radiologists. These might be overcome with a more automatic segmentation approach, such as deep learning frameworks. Third, previous studies have shown that multimodal ultrasound information is complementary and can improve diagnostic accuracy [44]. In further studies, multimodal ultrasound images, such as Doppler and elastic ultrasound, should be included.

In conclusion, the results of this multicenter study demonstrate that the ultrasound-based interpretable clinical-radiomics model enables non-invasive and accurate prediction of the three HER2 status classifications in breast cancer. Thus, the findings provide a potentially effective preoperative prediction tool for identifying patients who can benefit from HER2-targeted therapy.

## Supplementary information

ELECTRONIC SUPPLEMENTARY MATERIAL

## Data Availability

The datasets used and/or analyzed during the current study are available from the corresponding author upon reasonable request.

## Abbreviations

ALNM: Axillary lymph node metastasis
ANOVA: Analysis of variance
AUC: Area under the receiver operating characteristic curve
ER: Estrogen receptor
FISH: Fluorescence in situ hybridization
HER2: Human epidermal growth factor receptor 2
ICC: Intraclass correlation coefficient
IHC: Immunohistochemistry
LASSO: Least absolute shrinkage and selection operator
MRI: Magnetic resonance imaging
PR: Progesterone receptor
ROI: Region of interest
RS: Radiomics signature
SHAP: Shapley additive explanations
ZE: Zone entropy
