# Supplementary material for: Intratumoral and peritumoral ultrasound-based radiomics for preoperative prediction of HER2-low breast cancer: a multicenter retrospective study
Source: Insights Imaging. 2025 Mar 7;16:53. doi: 10.1186/s13244-025-01934-6 (PMC11889314; doi:10.1186/s13244-025-01934-6)
Supplement: Supplementary file 1 — ELECTRONIC SUPPLEMENTARY MATERIAL [file 13244_2025_1934_MOESM1_ESM.pdf]

Intratumoral and peritumoral ultrasound-based radiomics for preoperative prediction of HER2-low breast cancer: a multicenter retrospective study

ELECTRONIC SUPPLEMENTARY MATERIAL

Table S1. Details of ultrasound devices used in the study.

Manufacturer	Model	Production country	Probe	Frequency (MHZ)
Siemens Healthineers	OXANA2	Germany	L12-5	5-12
Siemens Healthineers	Acuson S1000	Germany	12L4	7-12
Siemens Healthineers	Acuson S2000	Germany	12L4	7-12
Siemens Healthineers	Seguoia 512	Germany	12L4	7-12
Hitachi Healthcare	Hitachi ALOKA Arietta 850	Japan	EUP-L53L	5-13
Hitachi Healthcare	Prerius	Japan	EUP-L53L	5-13
Toshiba Medical Systems	Aplio 500	Japan	PLT-1204BT	5-14
Toshiba Medical Systems	Aplio 400	Japan	PLT-1204BT	5-14
General Electric	LOGIQ E10S	American	GE 12L	7-12
General Electric	LOGIQ S7	American	GE 12L	7-12
Samsung Medison	RS80A	Korea	L7-12A	7-12
Mindray	MX7	China	L14-6	6-14
Mindray	Resona 8	China	L12-3E	3-12
Mindray	I9S	China	L14-6E	6-14
Esaote	MyLab 30	Italy	LA523	3-12

S1. Ultrasound assessment of axillary lymph node metastasis was negative unless one of the following criteria was met: 1) the long/short diameter ratio of axillary lymph node (ALN) <2; 2) diffuse or irregular thickening of the lymph node cortex >3 mm; 3) irregular hyperechoic area or microcalcifications in the ALN cortex; 4) complete or partial effacement of the echogenic fatty hilum; 5) replacement of ALN with an ill-defined or irregular hypoechoic mass; and 6) nonhilar cortical blood flow on color Doppler images, which is the appearance of peripheral vascular flow at the cortex of the node with no detectable connection to the hilum [1-4].

S2. Seven feature classes were extracted from each region of interest (ROI) in every image including first-order, shape, gray-level co-occurrence matrix (GLCM), gray-level size zone matrix (GLSZM), gray-level run length matrix (GLRLM), gray-level dependence matrix (GLDM), and neighboring gray tone difference matrix (NGTDM).

S3. Least absolute shrinkage and selection operator (LASSO) regression method: we set elastic net parameter (α) to 1, and a fivefold cross-validation was used to find the minimum mean squared error and determine the optimal regulation weight (λ). We set the correlation threshold at 0.6 to reduce feature redundancy. If the correlation between two features exceeds 0.6, remove the feature with a higher average correlation with the remaining features.

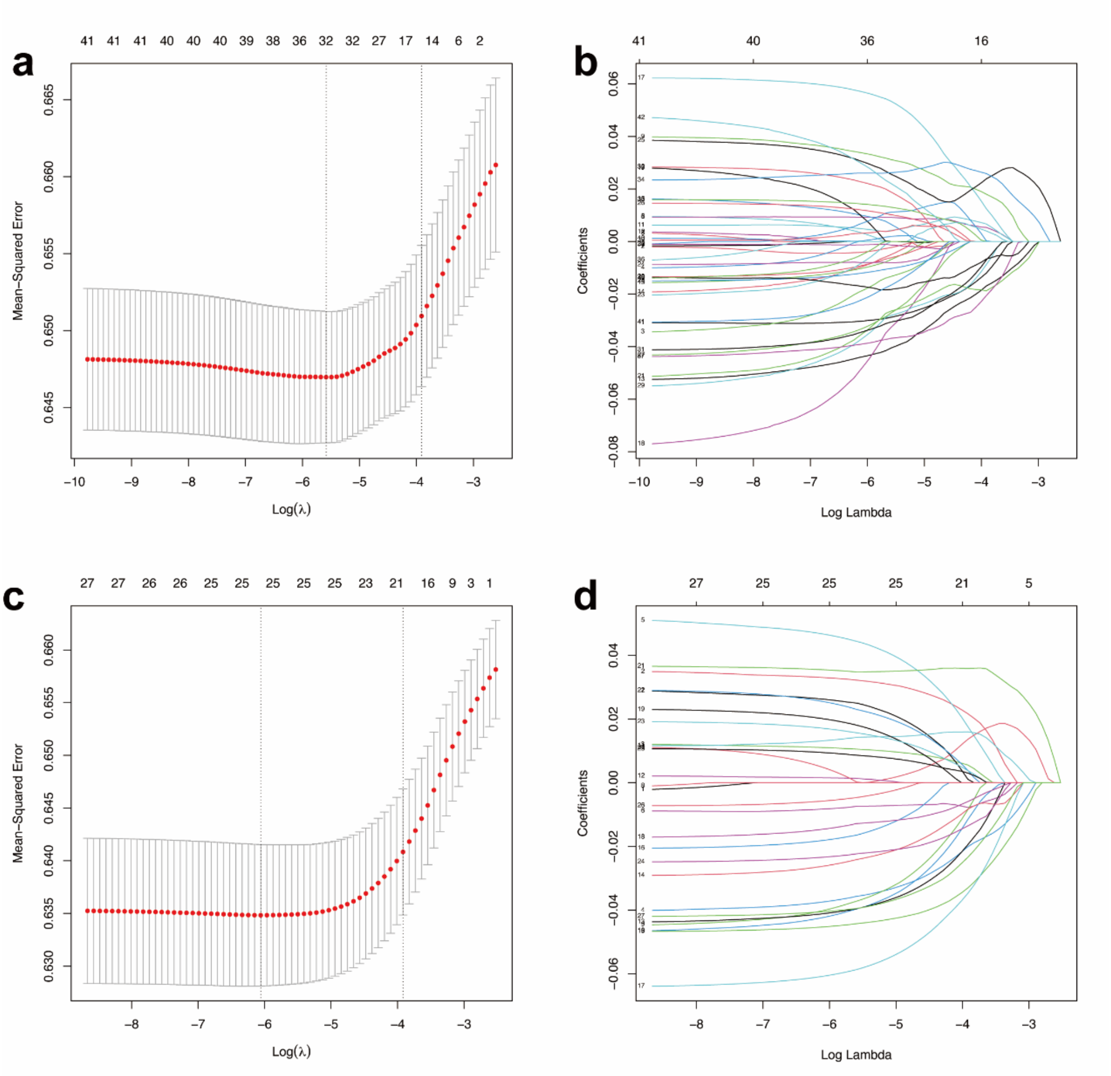

Figure S1. Feature selection using LASSO logistic regression in single-region and multi-region, take the Tumor and Tumor +Peri15mm models for instance. **a, c** Tuning parameters (λ) selection using cross-validation in Tumor and Tumor +Peri15mm models, respectively. **b, d** LASSO coefficient profiles of the features in Tumor and Tumor +Peri15mm models, respectively. LASSO, least absolute shrinkage and selection operator.

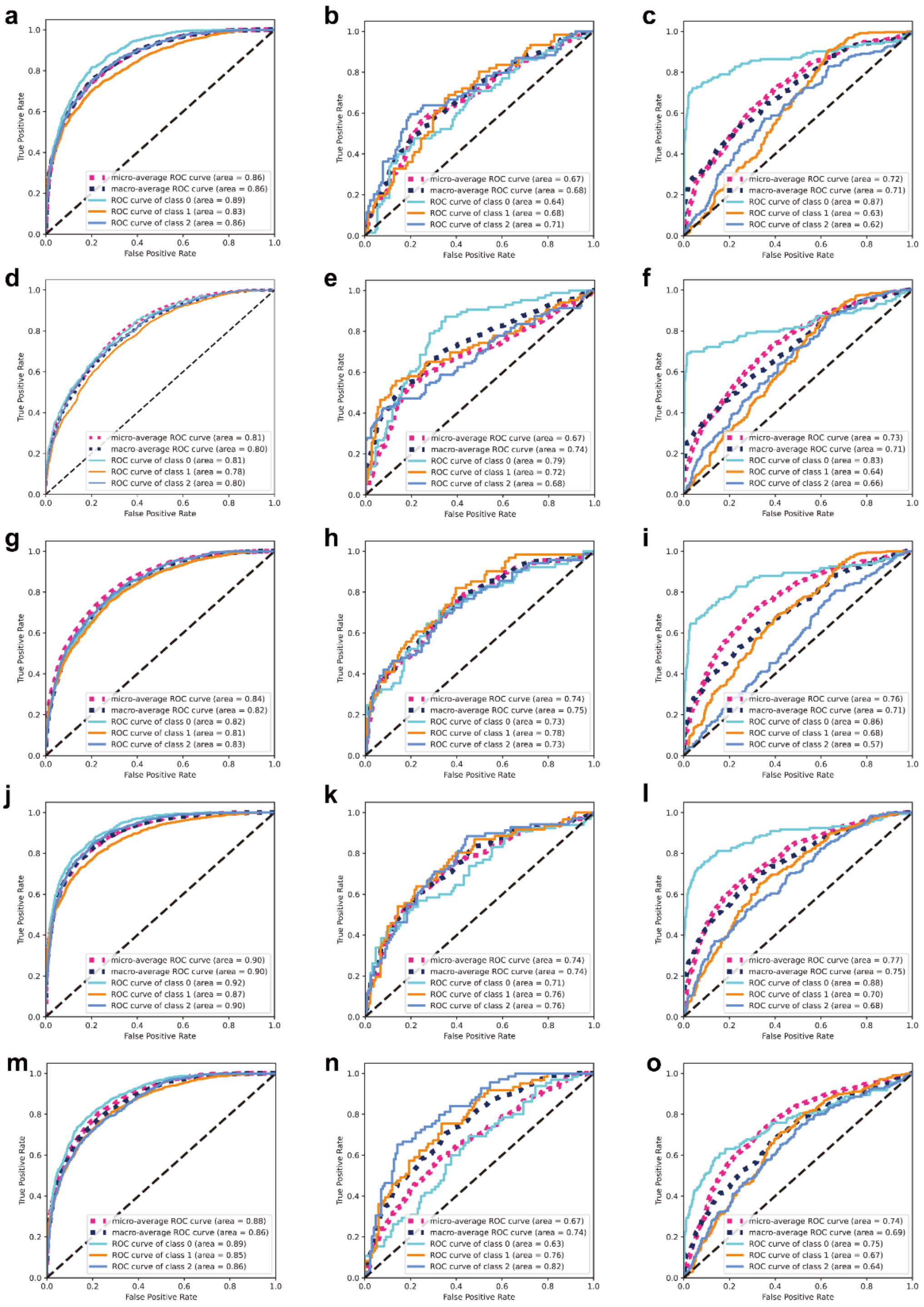

Figure S2. Performance of Tumor and four multi-region radiomics models in the training, validation, and test cohorts, respectively. **a-c** The ROC curves of Tumor model. **d-f** The ROC curves of Tumor +Peri5mm model. **g-i** The ROC curves of Tumor +Peri10mm model. **j-l** The ROC curves of Tumor +Peri15mm model. **m-o** The ROC curves of Tumor +Peri20mm model. 0 represents HER2-zero status, 1 represents HER2-low status, 2 represents HER2-positive status. ROC, receiver operating characteristic.

Table S2. Multinomial logistic regression analysis of various clinical and qualitative ultrasound factors influencing HER2 status.

Variables	HER2-low (Reference: HER2-zero)		HER2-positive (Reference: HER2-zero)		
	**coefficient**	**OR**	**coefficient**	**OR**	***p*-value**
(Intercept)	-0.643	0.526	-1.68	0.186	
Age	0.015	1.015	0.011	1.011	<0.001*
Size	-0.014	0.987	0.17	1.185	<0.001*
Tumor position	0.025	1.025	0.005	1.005	0.92
Shape	1.296	3.654	-12.249	0	<0.001*
Orientation	-0.07	0.933	0.32	1.377	<0.001*
Margin	-0.46	0.631	-0.525	0.591	0.019*
Echo pattern	-0.315	0.73	-0.175	0.839	0.002*
Posterior features	-0.048	0.953	-0.072	0.93	0.092
Calcifications	0.504	1.655	0.992	2.695	<0.001*
Edema	-0.044	0.957	-0.366	0.694	<0.001*
Skin change	0.315	1.37	0.1	1.105	0.009*
Vascularity	-0.165	0.848	-0.119	0.888	0.077

**p*<0.05. OR, Odds ratio.

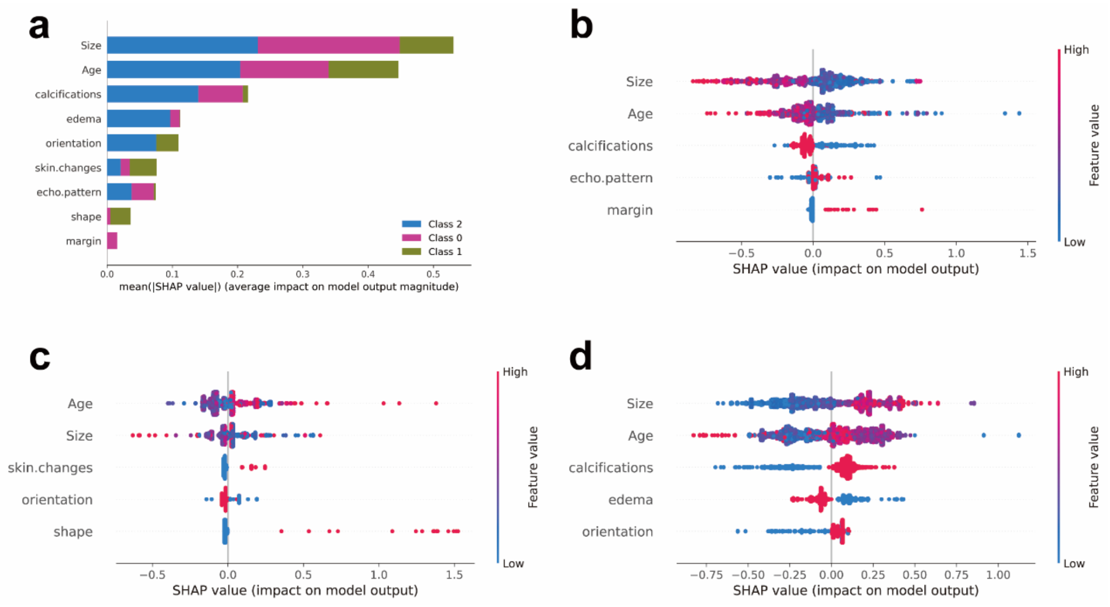

Figure S3 Features' contribution of the image-level clinical model. **a** The SHAP bar chart shows the importance of each feature for constructing the clinical model based on the mean SHAP values. **b-d** The SHAP summary plot shows the impact of top five features on the model predictions for different HER2 classes. **b** Prediction for HER2-zero class. **c** Prediction for HER2-low class. **d** Prediction for HER2-positive class. SHAP, Shapley additive explanations.

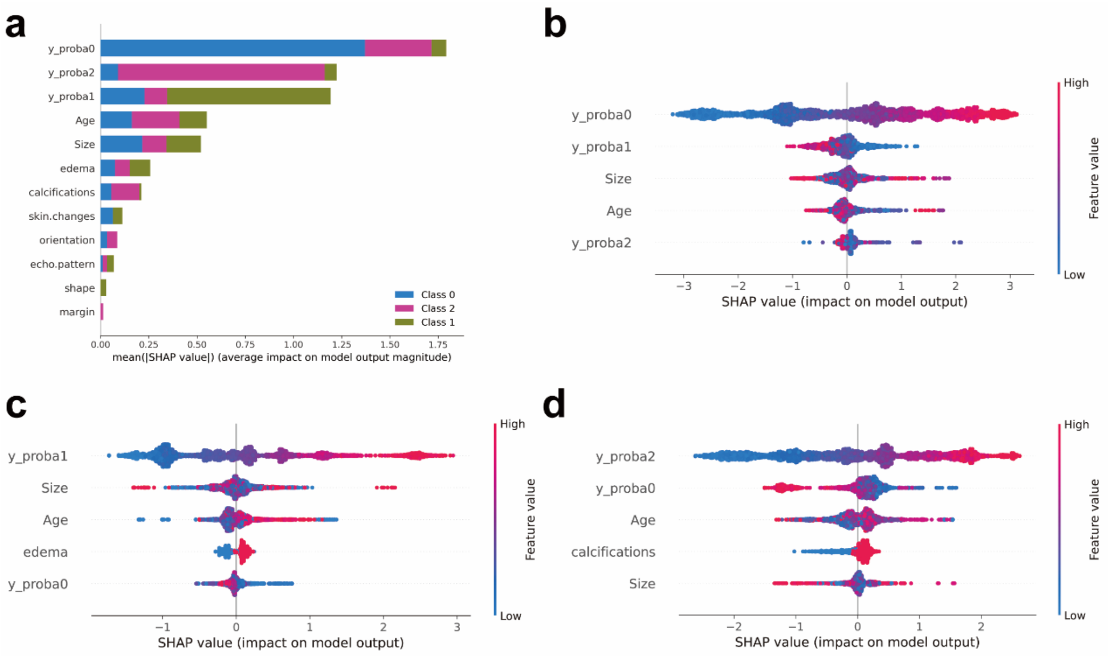

Figure S4. Features' contribution of the image-level clinical-radiomics combined model. **a** The SHAP bar chart shows the importance of each feature for constructing the combined model based on the mean SHAP values. **b-d** The SHAP summary plot shows the impact of top five features on the model predictions for different HER2 classes. **b** Prediction for HER2-zero class. **c** Prediction for HER2-low class. **d** Prediction for HER2-positive class. 0 represents HER2-zero status, 1 represents HER2-low status, 2 represents HER2-positive status. y_proba, prediction probability of radiomics model; SHAP, Shapley additive explanations.

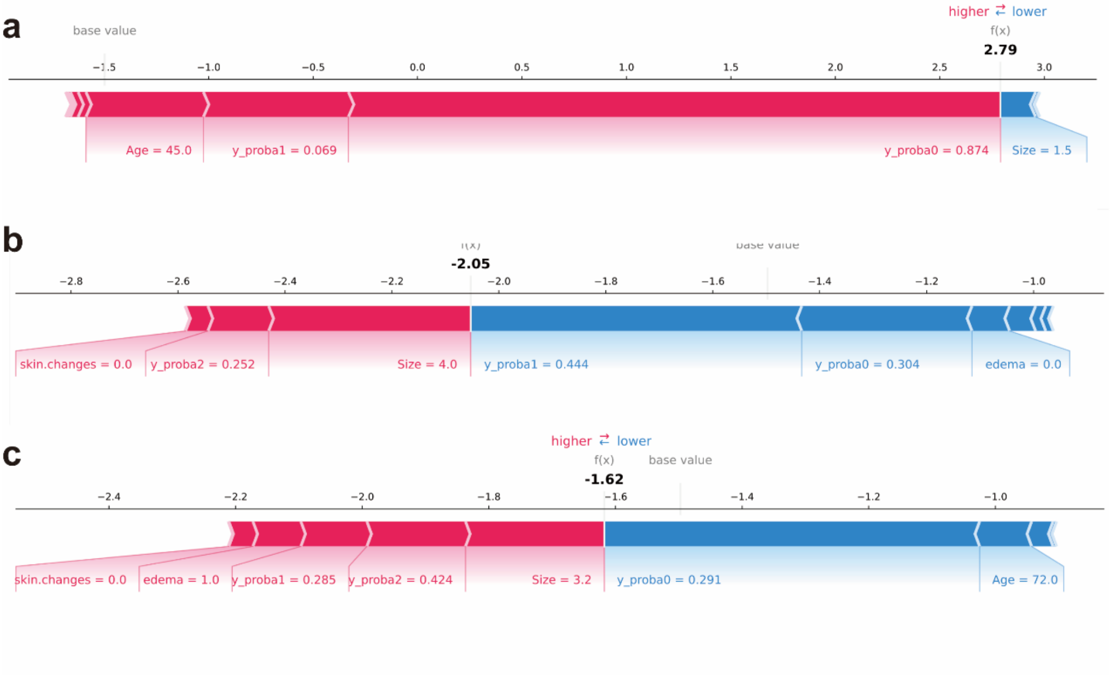

Figure S5. Application analysis of image-level clinical-radiomics combined model for three representative patients with different HER2 status. **a** HER2-zero patient. **b** HER2-low patient. **c** HER2-positive patient. 0 represents HER2-zero status, 1 represents HER2-low status, 2 represents HER2-positive status. y_proba, prediction probability of radiomics model.

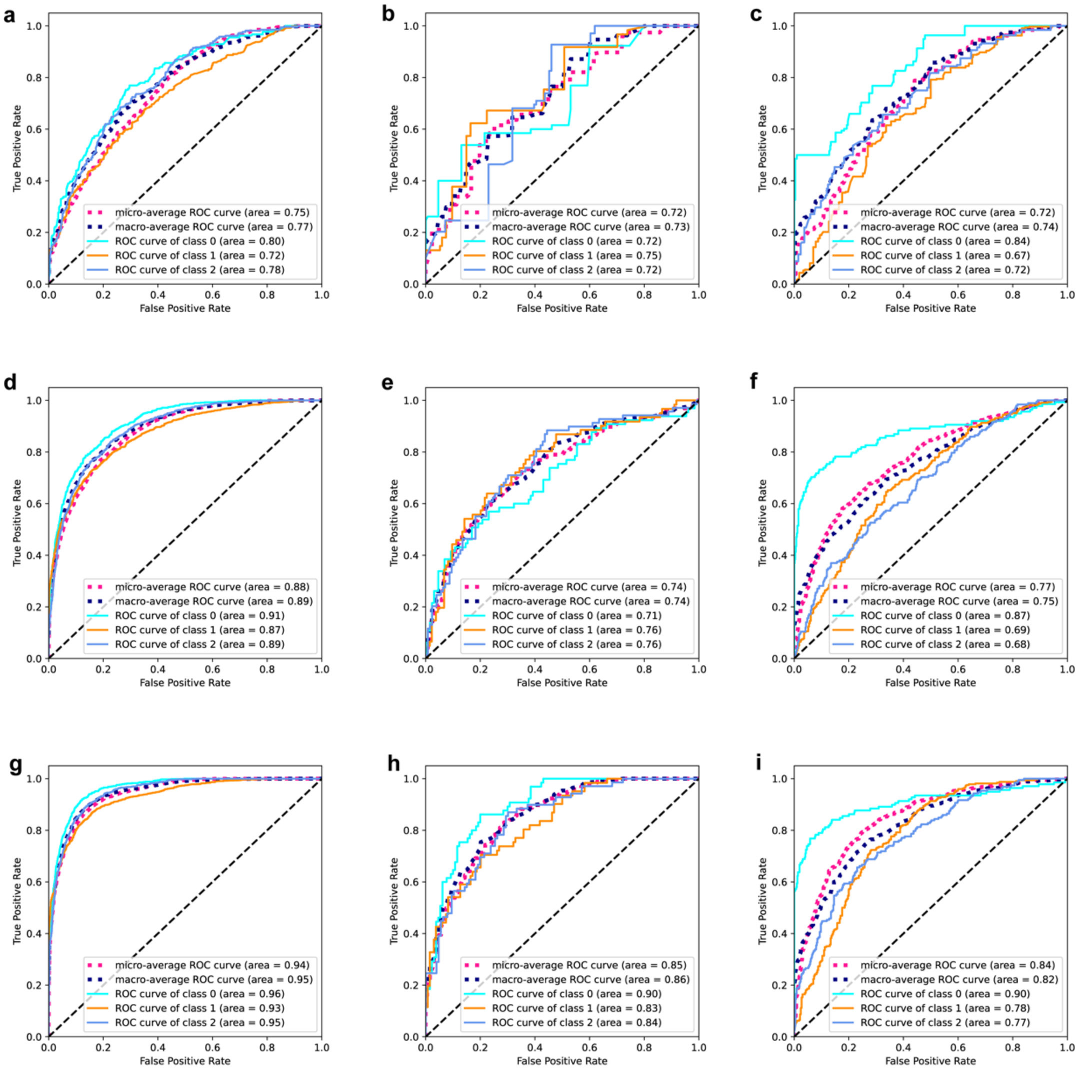

Figure S6. Performance of image-level models in the training, validation, and test cohorts, respectively. **a-c** The ROC curves of clinical model. **d-f** The ROC curves of radiomics model. **g-i** The ROC curves of combined model. 0 represents HER2-zero status, 1 represents HER2-low status, 2 represents HER2-positive status. ROC, receiver operating characteristic.

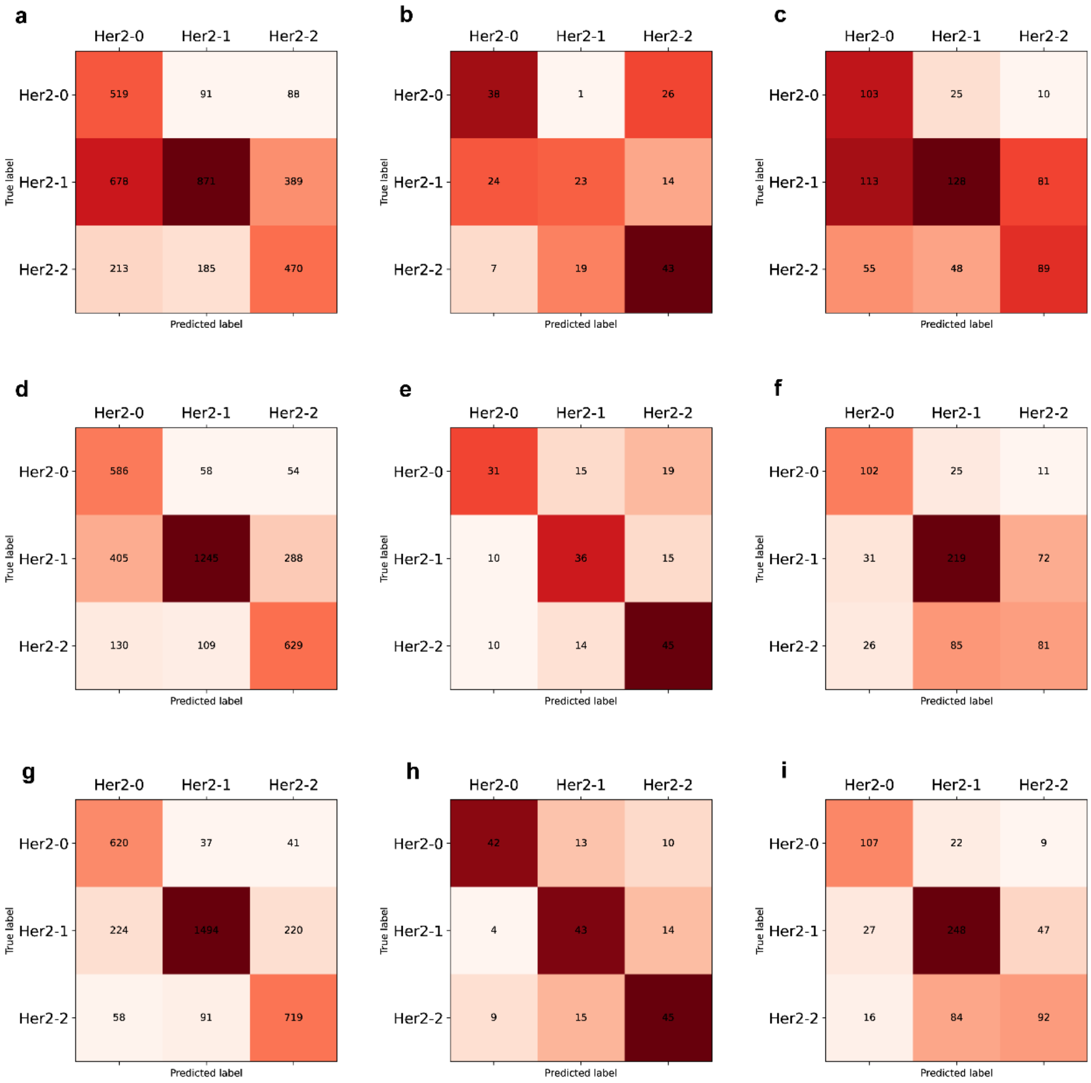

Figure S7. Confusion matrix of image-level models in the training, validation, and test cohorts, respectively. **a-c** clinical model, **d-f** radiomics model, **g-i** combined model. 0 represents HER2-zero status, 1 represents HER2-low status, 2 represents HER2-positive status.

References

1.Wang H, Yang XW, Chen F et al (2023) Non-invasive Assessment of Axillary Lymph Node Metastasis Risk in Early Invasive Breast Cancer Adopting Automated Breast Volume Scanning-Based Radiomics Nomogram: A Multicenter Study. Ultrasound in medicine & biology 49:1202-1211
2.Zheng X, Yao Z, Huang Y et al (2020) Deep learning radiomics can predict axillary lymph node status in early-stage breast cancer. Nat Commun 11:1236
3.Ecanow JS, Abe H, Newstead GM, Ecanow DB, Jeske JM (2013) Axillary staging of breast cancer- what the radiologist should know. Radiographics 33:1589-1612
4.Yang WT, Chang J, Metreweli C (2000) Patients with breast cancer- differences in color Doppler flow and gray-scale US features of benign and malignant axillary lymph nodes. Radiology 215:568-573
